# Macrophages Are a Double-Edged Sword: Molecular Crosstalk between Tumor-Associated Macrophages and Cancer Stem Cells

**DOI:** 10.3390/biom12060850

**Published:** 2022-06-19

**Authors:** Shahang Luo, Guanghui Yang, Peng Ye, Nengqi Cao, Xiaoxia Chi, Wen-Hao Yang, Xiuwen Yan

**Affiliations:** 1 Key Laboratory of Cell Homeostasis and Cancer Research of Guangdong Higher Education Institutes and Affiliated Cancer Hospital & Institute, Guangzhou Medical University, Guangzhou 910095, China; shahangluo@stu.gzhmu.edu.cn (S.L.); guanghuiyang@stu.gzhmu.edu.cn (G.Y.); 2019217972@stu.gzhmu.edu.cn (P.Y.); xiaoxia@stu.gzhmu.edu.cn (X.C.); 2 Department of Surgery, Nanjing Lishui People’s Hospital, Nanjing 211200, China; caonengqi@njslsormyy.wecom.work; 3 Graduate Institute of Biomedical Sciences, China Medical University, Taichung 406040, Taiwan

**Keywords:** cancer stem cells, tumor-associated macrophages, tumor microenvironment, cancer immunotherapy

## Abstract

Cancer stem cells (CSCs) are a subset of highly tumorigenic cells in tumors. They have enhanced self-renewal properties, are usually chemo-radioresistant, and can promote tumor recurrence and metastasis. They can recruit macrophages into the tumor microenvironment and differentiate them into tumor-associated macrophages (TAMs). TAMs maintain CSC stemness and construct niches that are favorable for CSC survival. However, how CSCs and TAMs interact is not completely understood. An understanding on these mechanisms can provide additional targeting strategies for eliminating CSCs. In this review, we comprehensively summarize the reported mechanisms of crosstalk between CSCs and TAMs and update the related signaling pathways involved in tumor progression. In addition, we discuss potential therapies targeting CSC–TAM interaction, including targeting macrophage recruitment and polarization by CSCs and inhibiting the TAM-induced promotion of CSC stemness. This review also provides the perspective on the major challenge for developing potential therapeutic strategies to overcome CSC-TAM crosstalk.

## 1. Introduction

Cancer stem cells (CSCs), also called tumor initiation cells, are specialized tumor cells that promote tumor initiation and progression [1]. The existence of CSCs was first confirmed in 1994 when Dick et al. grouped acute myeloid leukemia (AML) cells according to the expression of cell surface markers; when AML with CD34^+^CD38^−^ surface markers were transplanted into severe combined immunodeficiency (SCID) mice, numerous progenitor cell colonies were formed. However, non-CD34^+^CD38^−^ AML cells had limited proliferative capacity. Therefore, the CD34^+^CD38^−^ population was considered to be CSCs [2]. Since then, CSCs have been reported in other cancers: breast cancer (CSC markers: CD44, CD24, and ALDH-1) [3,4], colon cancer (CSC markers: CD24, CD44, CD133, and LGR5) [5,6,7], and melanoma (CSC markers: CD34 and ABCB5) [8,9]. CSCs are characterized by their self-renewal and differentiation potential that contribute to chemo-radioresistant and tumor initiation, which primarily account for the failure of current anticancer therapies in CSC-containing cancers [4]. In addition, CSC stemness is maintained mainly through the CSC niche, which is conducive to CSC survival, thereby making it difficult for drugs or other therapeutic methods to completely kill them, which leads to tumor recurrence [10]. Therefore, the eradication of CSCs is considered a promising method for improving cancer survival rates and even for curing cancer.

The CSC niche is a part of the tumor microenvironment (TME) and is similar to the normal adult stem cell niche that regulates the biological activity of stem cells in the form of cell–cell contacts and secreted factors [11,12]. The TME comprises fibroblasts, immune cells, endothelial cells, perivascular cells, extracellular matrix (ECM) components, and cytokine networks, among which tumor-associated macrophages (TAMs) are the most abundant immune cells in the TME [11]. TAMs can be recruited by CSCs and participate in TME formation; hence, they are beneficial to CSC survival [13]. Some studies have reported using CSCs derived from human or murine cholangiocarcinoma, hepatocellular carcinoma (HCC), or glioblastoma (GBM) cells; under in vitro spheroid culture, many factors regulate monocytes/macrophages in the supernatant, including CC-motif chemokine ligand (CCL) 2, CCL5, colony-stimulating factor (CSF)-1, interleukin (IL)-13, transforming growth factor (TGF)-β, and periosteum proteins (POSTN). These cytokines recruit circulating monocytes as well as surrounding tissue macrophage precursors into the TME and polarize them into TAMs [14,15,16]. After recruiting macrophages into the TME, CSCs can avoid phagocytosis by macrophages through protective mechanisms [17]. In clinical studies, CD47 expression has been reported to be upregulated in CSCs of HCC [18], pancreatic ductal adenocarcinoma [19], and lung cancer [20]. CD47 interacts with signal regulatory protein α (SIRP-α), which is expressed on TAMs, to prevent CSCs from being phagocytosed. Macrophages recruited into the TME can be divided into classically activated M1 macrophages and alternatively activated M2 macrophages [21]. M1 macrophages are induced and activated by interferon (IFN)-γ and lipopolysaccharides. Activated M1 macrophages typically express CD40 and CD86 and secrete higher levels of proinflammatory factors than M2 macrophages, such as IL-1α, tumor necrosis factor (TNF), and IL-12, which mainly exert an antitumor immune response and directly kill tumor cells in the early stage of a tumor [22]. Therefore, injecting M1 macrophages into the tumor tissue may be a potential therapeutic strategy for cancer [23], but the specific mechanism of action remains unclear. Activated M2 macrophages, however, play a role opposite to that of M1 macrophages—promoting ECM remodeling and tumor growth and metastasis [24]. M2 macrophages support CSC signatures, and consequently, they are also called M2 TAMs [25]. They are induced by IL-4 and IL-13, often with CD163 and CD206 as markers [26]. M2 macrophages can secrete soluble mediators, such as platelet-derived growth factor (PDGF), vascular epidermal growth factor (VEGF), and cytokines, such as IL-6, IL-10, and TNF-α [15,27,28]. These soluble mediators and cytokines can maintain the stemness characteristics of tumor cells by regulating the nuclear factor-κB (NF-κB), signal transducer and activator of transcription (STAT)3, AKT, and Hippo pathways [14,27,29,30,31,32]. These findings highlight the importance of molecular crosstalk between CSCs and macrophages in tumor progression; targeting these molecular mechanisms may provide more effective immunotherapies.

The CSC niche provides favorable conditions for drug resistance, tumor recurrence, and metastasis. TAMs, as the main immune cells in the TME, also play a crucial role in cancer malignancy. In this review, we summarize the potential mechanisms of interaction between CSCs and TAMs and discuss the current molecular targeting studies thereof. An overview of these emerging insights can provide new directions for antitumor strategies.

## 2. Secretory Molecules of CSCs Induce Macrophage Recruitment and Tumor-Promoting Characteristics of the TME

**Figure 1:**
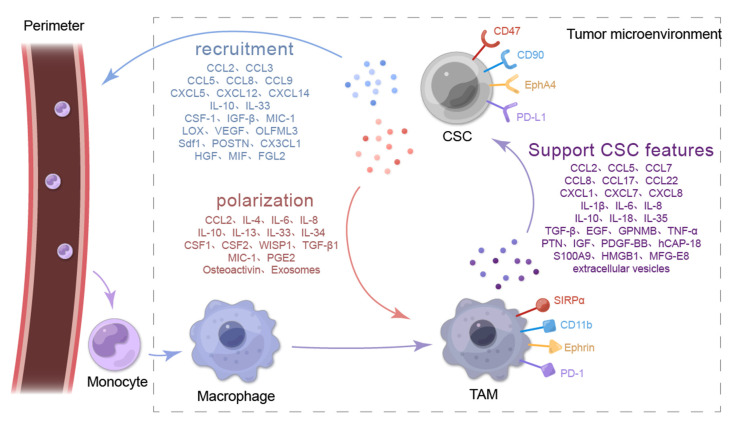
Molecular crosstalk between TAMs and CSCs. CSCs promote macrophage recruitment by secreting CCL2, CCL3, CCL5, CCL8, CCL9, CXCL5, CXCL12, CXCL14, IL-10, IL-33, CSF-1, IGF-β, MIC-1, LOX, VEGF, OLFML3, Sdf1, POSTN, CX3CL1, HGF, MIF, and FGL2. CSCs promote macrophage polarization by secreting CCL2, IL-4, IL-6, IL-8, IL-10, IL-13, IL-33, IL-34, CSF1, CSF2, WISP1, TGF-β1, MIC-1, PGE2, Osteoactivin, and exosomes. TAMs produce factors to support CSC-like features, including CCL2, CCL5, CCL7, CCL8, CCL17, CCL22, CXCL1, CXCL7, CXCL8, IL-1β, IL-6, IL-8, IL-10, IL-18, IL-35, TGF-β, EGF, GPNMB, TNF-α, PTN, IGF, PDGF-BB, hCAP-18, S100A9, HMGB1, MFG-E8, and extracellular vesicles.

The functions of CSCs, such as immune escape, therapy resistance, metastasis, and maintenance of stemness, are largely dependent on the secretion of soluble factors (chemokines, interleukins, growth factors, or secreted proteins) and extracellular vesicles [33,34,35]. Specifically, CSCs signal by secreting soluble factors to recruit peripheral macrophages to the TME to become part of the tumor niche and by inducing macrophage transformation into TAMs [36]. A comprehensive understanding of the underlying mechanisms of macrophage recruitment and polarization by CSCs is beneficial for determining how to interrupt the mutual support pathway between CSCs and TAMs. Figure 1 presents the molecular interaction mechanisms between TAMs and CSCs.

### 2.1. CSCs Recruit Macrophages into the TME

Many cytokines secreted by tumor cells can recruit macrophages to the TME, but some of them are exclusively produced by CSCs: CCL2, CCL3, CCL5, CXC motif chemokine ligand 12 (CXCL12), olfactomedin-like 3 (OLFML3), stromal-cell-derived factor-1b (Sdf1b), IL-6, IL-33, and CSF-1 [37,38,39,40,41,42,43,44,45] (Figure 1). CSCs contribute to the infiltration of macrophages or microglia to promote their own metastatic spread [38,39,43,46]. Here, we summarize the cytokines and soluble protein molecules secreted for macrophage recruitment by CSCs in different tumor types (Table 1).

**Table 1:** CSCs secrete cytokines and other soluble protein molecules to recruit macrophages.

Chemokines Produced by CSCs	Chemokine Receptor	Type of Cancer	Associated Signaling Pathway/Mechanism of Chemokine Secretion	Reference
CCL2 ^1^	CCR2	Glioblastoma	IFNγ	[47]
		Breast cancer	β-Catenin	[48]
		Bladder cancer	Not shown	[39]
		Hepatocellular carcinoma	Hippo	[43]
CCL3	CCR1/CCR5	Bladder cancer	Not shown	[49,50]
		Leukemia	Not shown	[51]
CCL5	CCR5, CCR1, CCR2	Glioblastoma	PI3K ^2^/AKT	[52,53]
		Optic glioma	Not shown	[54]
		Hepatocellular carcinoma	Not shown	[55]
CCL8	CCR2	Bladder cancer	Not shown	[56]
		Cutaneous squamous cell carcinoma	Not shown	[57]
CCL9	CCR1	Non-small cell lung cancer	Not shown	[58]
CXCL5 ^3^	CXCR2	Hepatocellular carcinoma	Sox9	[59]
CXCL12	CXCR4	Leukemia	Not shown	[42]
		Colon cancer	NF-κB ^4^	[60]
CXCL14	Not shown	Lung cancer	Not shown	[61]
IL-10	IL-10R	Ovarian cancer	WNT	[62]
IL-33	ST2	Squamous cell carcinoma	NRF2	[44]
CSF1 ^5^	CSF1R	Hepatocellular carcinoma	Hippo	[43]
		Non-small cell lung cancer	IRF5	[63]
		Glioblastoma	STAT3 ^6^	[64]
TGF-β ^7^	Not shown	Glioblastoma	STAT3	[64]
MIC-1 ^8^	Not shown	Glioblastoma	Not shown	[64]
		Pancreatic cancer	Not shown	[65]
LOX ^9^	Not shown	Pancreatic cancer	Not shown	[66]
VEGF ^10^	VEGFR	Colorectal cancer	Six1/MAPK ^11^ & PI3K/AKT/mTOR	[67,68]
OLFML3 ^12^	Not shown	Glioblastoma	CLOCK-BMAL1	[40]
Sdf1 ^13^	Cxcr4b	Brain tumor	AKT1	[41]
POSTN ^14^	Integrinαvβ₃	Glioblastoma	Not shown	[69]
CX3CL1	CX3CR1	Testicular germ cell tumors	Not shown	[70]
HGF ^15^	Not shown	Glioblastoma	HGF-MET axis	[71]
MIF	CXCR4	Glioblastoma	Not shown	[72]
FGL2	Not shown	Glioblastoma	Not shown	[73]

^1^ C-C-motif chemokine ligand 2; ^2^ Phosphoinositide 3-kinase; ^3^ CXC motif chemokine ligand 5; ^4^ Nuclear factor-kappa B; ^5^ Colony-stimulating factor-1; ^6^ Signal transducer and activator of transcription (STAT)3; ^7^ Transforming growth factor β; ^8^ Macrophage inhibitory cytokine-1; ^9^ Lipoxygenase; ^10^ Vascular epidermal growth factor; ^11^ Mitogen-activated protein kinase; ^12^ Olfactomedin like 3; ^13^ Stromal-cell-derived factor-1b; ^14^ Periostin; ^15^ Hepatocyte growth factor.

#### 2.1.1. CSCs Recruit Macrophages through the CC Chemokine Family and the CXC Chemokine Subfamily

The CC chemokine family and the CXC chemokine subfamily are involved in macrophage recruitment in tumors (or CSCs) [74]. CCL2, a CC chemokine, is secreted by CSCs of different tumor types to promote macrophage infiltration. CCL2 overexpression is associated with poor patient prognosis in various tumor types [75] (Table 1). In studies on GBM [38], prostate cancer [76], and bladder cancer [39], CCL2 expression was significantly increased in CSCs compared with non-CSCs, and the macrophage count in the TME was also increased. Another study showed that the blockade of CCL2 binding to its receptor CCR2 on macrophages or depletion of CCL2 decreased monocyte recruitment and prolonged tumor survival in mice [43]. Thus, CCL2 may play a vital role in macrophage recruitment. Studies have attempted to uncover why CCL2 is upregulated in CSCs. In a study of liver CSCs, CCL2 expression was associated with Yes-associated protein, which acts as a transcriptional coactivator and can upregulate CCL2 expression [43]. Furthermore, the upregulation of CCL2 is due to the increase of the stemness-related transcription factor TWIST, and the increased CCL2 secretion promotes the infiltration of M2 macrophages in lung cancer [77]. GBM stem cells (GSCs) have stable epigenetic changes that initiate a myeloid-dependent transcriptional program that expresses myeloid-specific master transcription factors such as interferon regulatory factor (IRF)1 and IRF8 [47]. Together, these findings reveal that CSCs may induce macrophage recruitment by secreting CCL2; however, the underlying mechanism remains unclear.

CCL3, also known as macrophage inflammatory protein-1α, is a ligand for the receptors CCR1 and CCR5 (Figure 1) and promotes macrophage recruitment [78]. CCL3 is secreted in CD14^+^ bladder CSCs rather than CD14^−^ cancer cells, and macrophage infiltration is also increased in the TME [49]. However, another study determined that CCL3 recruits macrophages to the TME to exert anticancer activities [79]. These results indicate that CSCs can secrete CCL3 to recruit macrophages with antitumor properties in the TME.

Other chemokine members also participate in macrophage recruitment. In a study on GBM, CCL5 expression was significantly higher in GSCs compared with low-grade gliomas and noncancerous tissues, whereas GSCs did not express CCR5 (Figure 1). GSCs may secrete CCL5, which binds to microglial CCR5 and causes microglial infiltration [52,53]. Microglia are known as the macrophages of the nervous system, and their biological properties are similar to those of peripheral macrophages [80]. Notably, CCL5 is produced exclusively by CSCs, highlighting their unique role in macrophage recruitment. Furthermore, the ability to recruit microglia through CCL5 is enhanced when PTEN or FGFR1 is mutated [53]. By contrast, a study reported that CCL8 secreted by CSCs can recruit M1 macrophages and increase the number of CD16 and CD80 macrophages in the TME [57]. However, the study did not explain why CSCs recruited M1 macrophages, which are thought to have antitumor properties. In fact, tumor stromal mesenchymal stem cells (MSCs) in the TME are also involved in macrophage recruitment, and MSCs highly express various chemokines, especially CCL2, which increase the recruitment of monocytes/macrophages to the TME [81]. In conclusion, accumulating evidence indicates that the CC family of chemokines secreted by the CSCs of different cancer types are involved in macrophage recruitment.

Chemokines in the CXC family secreted by CSCs are also involved in macrophage recruitment. CXCL5 expression is higher in CD44^+^ CSCs compared with non-CSCs [82]. CXCL5 acts as a key downstream mediator of Sox9, which is a biomarker of liver CSCs. Sox9 can bind to the promoter of CXCL5 to induce CXCL5 secretion, and a high CXCL5 level enhances macrophage recruitment in HCC [59]. Furthermore, the CXCL12 level has a significant positive correlation with macrophage infiltration in tumors [60]. CD133^+^ GCSC can also secrete CXCL12, which binds to CXCR4 (CXCL12 receptor) on macrophages to recruit macrophages to the TME [83]. Similar results were also observed in breast cancer [84]. These data suggest that both the CC and CXC chemokine families secreted by CSCs play a crucial role in macrophage recruitment. Targeting these chemokines may inhibit macrophage recruitment by CSCs, thereby hindering CSC niche formation.

#### 2.1.2. CSCs Recruit Macrophages by Secreting IL-33

CSCs recruit and induce myeloid cells to differentiate into TAMs by releasing IL-33 [85] (Figure 1). IL-33 combined with ST2 on TAMs stimulates TAMs to secrete TGF-β. TGF-β can regulate CSCs through a negative feedback mechanism, activate NRF2-mediated antioxidant responses, and release IL-33, thus promoting macrophage recruitment [44]. This suggests that macrophage recruitment by CSCs is an interactive process. Interruption of the IL-33–TGF-β signaling loop provides a potential therapeutic option for preventing CSC niche formation.

#### 2.1.3. CSCs Recruit Macrophages through Other Types of Secreted Proteins

In addition to chemokines and ILs, CSCs can also secrete various other soluble cellular proteins to promote macrophage recruitment into the TME, such as CSF-1 [86] (Figure 1). Yamashina et al. reported that CSCs stimulated by interferon regulatory factor 5 (IRF5) could activate the IRF5/CSF-1 pathway in non-small-cell lung cancer (NSCLC), thereby increasing CSF-1 secretion and causing an increase in the number of M2 macrophages with CSF-1 receptors [63]. However, GSCs can produce CSF-1 and cytokines, such as TGF-β1 and macrophage inhibitory cytokine (MIC)-1, to recruit surrounding microglia [64]. These results suggest that CSCs promote macrophage recruitment by secreting these cytokines, but what drives CSCs to secrete these soluble cytokines is unclear. Targeting these cytokines may effectively block macrophage recruitment into the TME and prevent macrophages from participating in CSC niche formation.

CSCs can also promote macrophage recruitment by secreting other soluble proteins, including lipoxygenase (LOX), VEGF-A, OLFML3, IRF, Sdf1, and POSTN (Figure 1). In a mouse model of pancreatic cancer, Janakiram et al. demonstrated that LOX can also promote macrophage recruitment [66]. In a model of natural killer T cell loss, stimulation of 5-LOX expression in CSCs resulted in a synchronous increase in the number of M2-type macrophages, suggesting that LOX may be involved in macrophage recruitment [66]. In GBM, binding of the circadian regulator CLOCK and its heterodimeric partner BMAL1 upregulates the transcription of OLFML3, which acts as a chemokine and simultaneously recruits immunosuppressive microglia to the TME to enhance CSC stemness [40]. In the early stage of brain tumors, GSCs, marked by Sox2, were found to promote macrophage/microglia infiltration through the Sdf1b–CXCr4b signaling pathway, and in vivo confocal imaging revealed that macrophages/microglia and CSCs have highly dynamic interactions and do not lead to the phagocytosis of CSCs [41]. In GBM, CSCs secrete more POSTN than non-CSCs to recruit macrophages, and neutralization of POSTN can significantly reduce TAM density [87]. POSTN binds to integrin αvβ₃ receptors on macrophages to increase their recruitment, which can be inhibited by blocking this signaling with the RGD peptide [69]. These results reveal that CSCs promote macrophage recruitment by secreting several soluble proteins. Targeting these pathways can be a new strategy for cancer treatment.

### 2.2. CSCs Promote Macrophage Polarization in the TME

When macrophages infiltrate the TME, CSC-secreted cytokines can induce the polarization of macrophages into M2 TAMs. These cytokines include CC chemokines, the IL family, prostaglandin E2 (PGE2), WNT1-inducible signaling pathway protein 1 (WISP1), and exosomes, some of which are also involved in macrophage recruitment (Figure 1). Table 2 summarizes the CSC-secreted cytokines and soluble protein molecules that promote macrophage polarization in different tumor types.

**Table 2:** CSCs secreted molecules to induce the polarization of macrophages.

Chemokines Produced by CSCs	Chemokine Receptor	Type of Cancer	Associated Signaling Pathway/Mechanism of Cytokine Secretion	Reference
CCL2	CCR2	Breast cancer	β-catenin/CCL2 axis	[48]
IL-4	IL-4R	Breast cancer	ERK1/2 ^1^	[88]
IL-6	IL-6R	Glioblastoma	TLR4	[89]
		Triple negative breast cancer	HLF/MCT-1	[90,91]
IL-8	IL-8R	Ovarian cancer	STAT3	[92]
IL-10	IL-10R	Ovarian cancer	PPARγ ^2^/NF-κB	[93]
IL-13	IL-13R	Cholangiocarcinoma	Not shown	[94]
IL-34	IL-34R	Cholangiocarcinoma	Not shown	[94]
IL-33	ST-2	Squamous cell carcinoma	NRF2	[44]
CSF1	CSF1R	Glioblastoma	Not shown	[64]
		Non-small cell lung cancer	Oct4	[95]
		Breast cancer	Not shown	[96]
CSF2	CSFR2	Triple negative breast cancer	NF-κB	[97]
		Pancreatic cancer	Not shown	[98]
WISP1	Integrinα6β1	Glioblastoma	WNT/β-catenin	[99]
TGF-β	TGFBR	Glioblastoma	Not shown	[64]
		Pancreatic cancer	Not shown	[98]
		Hepatocellular carcinoma	TLR4	[100]
MIC-1	Not shown	Glioblastoma	Not shown	[64]
PGE2 ^3^	EP2	Ovarian cancer	COX-2 ^4^/PGE2	[101]
		Glioblastoma	ARS2 ^5^/MAGL	[102]
Osteoactivin	Not shown	Cholangiocarcinoma	Not shown	[94]
Exosomes	Not shown	Pancreatic cancer	Not shown	[103,104]
		Glioblastoma	Not shown	[105]
		Esophageal squamous cell carcinoma	Not shown	[106]

^1^ Extracellular signal-regulated protein kinases 1 and 2; ^2^ Peroxisome proliferator-activated receptor-γ; ^3^ Prostaglandin E2; ^4^ Cyclo-oxygenase 2; ^5^ Arsenite-resistance protein 2.

#### 2.2.1. CSCs Induce Macrophage Polarization by Secreting CC Chemokines

Some chemokines secreted by CSCs, including CCL2 and CCL5, can also promote macrophage polarization while recruiting macrophages. For example, breast CSCs (BCSCs) activate the β-catenin pathway to regulate CCL2 expression and promote macrophage polarization to the M2 type [48] (Table 2). In addition, CSCs promote macrophage polarization through the β-catenin/CCL2 axis. However, macrophages secrete CCL2 to maintain the characteristics of BCSCs [48]. Zhuang et al. reported that CCL5 can transform M0 macrophages (inactivated macrophages) into M2 TAMs when cocultured with hepatoma cells; the result is the expression of high levels of IL-10, IL-12, and TNF-α [55]. However, the mechanism by which activated TAMs secrete cytokines via the CCL5–CCR5 pathway remains unclear, thus necessitating further research.

#### 2.2.2. CSCs Promote Macrophage Polarization by Secreting ILs

To date, most studies support the idea that CSCs induce macrophage polarization in the TME by secreting ILs, including IL-6, IL-8, IL-10, IL-13, and IL-34 (Table 2). Weng et al. reported that MCT-1-overexpressing triple-negative breast cancer (TNBC) cells secrete IL-6, which activates monocytes/macrophages to an M2 TAM-like phenotype via the JAK2/STAT3 pathway and that this pathway can be inhibited after neutralization with IL-6 antibodies [90,91]. In liver cancer, M2 polarization of macrophages is also regulated via the IL-6/STAT3 pathway, the inhibition of which causes macrophages to be polarized to M1 [107]. In addition, CSCs may secrete IL-8 to promote macrophage polarization. IL-8 levels are increased when ovarian CSCs (OCSCs) are cocultured with macrophages [92]. OCSC can promote M2 polarization of macrophages via the IL-8/STAT3 signaling pathway. In addition, IL-10 is highly expressed in OCSCs and induces macrophage polarization toward M2 TAMs by activating the PPARγ/NF-κB pathway [93]. These findings suggest that CSCs can induce macrophage polarization in the TME by secreting ILs, but the specific underlying pathways remain unclear.

#### 2.2.3. CSCs Promote Macrophage Polarization through Other Cytokines and Soluble Protein Molecules

The cytokine CSF secreted by CSCs, which promotes macrophage recruitment, can also cause macrophage polarization. In pancreatic cancer, TGF-β1 and CSF-2 secreted by pancreatic CSCs (Pa-CSCs) were reported to be positively correlated with the levels of M2 TAM-related molecules. In addition, the use of TGF-β1 or CSF-2 inhibitors repressed the polarization of M2 TAMs, indicating that Pa-CSCs can promote macrophage polarization to M2 TAMs through TGF-β1 and CSF-2 [98]. High CSF-2 levels can be secreted by TNBC cells in an NF-κB-dependent manner to promote macrophage polarization into M2 macrophages [97]. CSF-1 can also promote macrophage polarization in a glioma model [64]. M1 macrophages promote lung cancer to express octamer 4 (Oct4) to upregulate CSF-1 gene expression, and CSF-1 stimulates the activation of M2 macrophages [95]. Notably, M1 macrophages induce CSF-1 gene expression in CSCs, thereby stimulating M2 TAM activation and extending our understanding of TAMs.

CSCs can also secrete prostaglandin F2 (PGF2), WISP1, and other soluble protein molecules to promote the activation of macrophages in the TME. In GSCs, PGF2, produced via the ARS2–MAGL signaling pathway, can induce the transformation of macrophages to M2 TAMs [102]. In addition, OCSCs secrete PGE2 via the COX-2/PGE2 pathway, which activates Janus kinase (JAK) signaling in M2 macrophages and significantly increases the secretion of IL-10 and CD206^+^ on M2 macrophages [101]. CSCs may activate the related pathway of M2 macrophage polarization by secreting PGF2. Furthermore, GSCs induce the secretion of WISP1 via the WNT pathway, supporting M2 polarization of macrophages in a paracrine manner. In addition, inhibition of WNT/β-catenin-WISP1 signaling can significantly reduce macrophage polarization [99]. To summarize, CSCs can transform macrophages into TAMs by secreting soluble proteins.

The direct contact of some proteins on CSCs and TAMs can also promote macrophage polarization. CSCs can bind phosphatidylserine to apoptotic cell membranes by binding to receptor tyrosine kinase family protein molecules (Tyro3, Axl, and MerTK) on TAMs using bridging ligand Gas6 and protein S, and post-efferocytosis, macrophages are polarized to an M2-like phenotype [108]. This finding suggests that CSCs are involved in macrophage transformation into TAMs by secreting these proteins in different tumor types. More studies on macrophage transformation into TAMs are warranted to reveal the immunosuppressive mechanisms in malignant tumors, and these pathways will provide reliable ideas for future tumor therapy.

#### 2.2.4. CSCs Polarize Macrophages by Secreting Exosomes

CSCs can also induce macrophage transformation into TAMs by producing exosomes in the TME [103,104]. GSC-derived exosomes can enter monocytes to reconstitute the actin cytoskeleton, thereby inducing their transformation into M2 TAMs [105]. The same result was also observed in esophageal squamous cell carcinoma [106]. In conclusion, CSCs may stimulate macrophage polarization by secreting exosomes and releasing miRNAs; this may provide a direction for the development of new biomarkers.

## 3. TAMs Enhance the Stemness Characteristics of Tumor Cells

The supporting role of TAMs in CSCs is crucial. After polarization, TAMs actively participate in CSC niche formation and play a crucial role in maintaining the self-renewal capacity of CSCs and tumor initiation. Notably, cytokines produced by TAMs can enable “differentiated” cancer cells to regain the characteristics of CSCs and maintain CSC stemness in tumors. This process of regaining the characteristics of CSCs transpires mainly through epithelial–mesenchymal transition (EMT) [109]. Table 3 summarizes the TAMs that support CSC stemness by secreting cytokines, soluble proteins, and exosomes in different tumor types.

**Table 3:** TAMs secreted molecules to enhance the properties of CSCs.

TAM Type	Factor of TAM Production	Type of Cancer	Associated Signaling Pathway/Mechanism of TAMs on CSCs	Reference
M2	CCL2	Breast cancer	β-catenin	[48]
	CCL5	Prostate cancer	β-catenin/STAT3	[110]
		Glioblastoma	Not shown	[52]
	CCL7	Ovarian cancer	CCR3/MM9 ^1^	[111]
	CCL8	Glioblastoma	CCR1/CCR5/ERK1/2	[112]
		Gastric cancer	JAK1/STAT3	[113]
	CCL17	Hepatocellular carcinoma	WNT/β-catenin	[114]
	CCL22	Hepatocellular carcinoma	Not shown	[115]
	CXCL1	Breast cancer	Not shown	[116]
	CXCL7	Glioblastoma	Not shown	[73]
	CXCL8	Breast cancer	CXCR2	[117]
	IL-1β	Glioblastoma	p38 MAPK	[118]
	IL-6	Glioblastoma	Not shown	[89]
		Ovarian cancer	WNT	[62]
		Hepatocellular carcinoma	STAT3	[119]
		Breast cancer	STAT3	[120]
	IL-8	Ovarian cancer	JAK2/STAT3	[92]
	IL-10	Ovarian cancer	WNT	[62]
		Non-small cell lung cancer	JAK1/STAT1/NF-κB/Notch1	[121]
	IL-18	Gastric cancer	Not shown	[122]
	IL-35	Breast cancer	Not shown	[123]
	TGF-β	Hepatocellular carcinoma	EMT	[124]
		Pancreatic cancer	TGF-β1/smad2/3 axis	[125]
		Squamous cell carcinoma	NRF2	[44]
		Lung cancer	Not shown	[126]
		Breast cancer	EMT	[127]
	TNF-α	Clear cell renal cell carcinoma	NF-κB	[128]
		Chordoma	PI3K/AKT	[129]
	EGF ^1^	Breast cancer	EGFR/STAT3/Sox2	[130]
	GPNMB ^2^	Breast cancer	PI3K/AKT/mTOR & β-catenin/MAPKs/AMPK/Src	[131]
	PTN ^3^	Lymphoma	PTPRZ1/β-catenin	[132]
		Glioblastoma	PTN-PTPRZ1	[133]
	IGF ^4^	Thyroid cancer	PI3K/AKT/mTOR	[134]
	PDGF-BB ^5^	Breast cancer	Not shown	[135]
	hCAP-18	Pancreatic ductal adenocarcinoma	Not shown	[136]
	S100A9 ^6^	Hepatocellular carcinoma	NF-κB	[137]
	HMGB1 ^7^	Non-small cell lung cancer	NF-κB	[138]
	MFG-E8 ^8^	Colorectal cancer	STAT3/Sonic Hedgehog	[139]
	extracellular vesicles	Pancreatic cancer	KLF3	[140]
M1	IL-6	Oral squamous cell carcinoma	JAK/STAT3	[141]
	IL-12	Glioblastoma	Not shown	[142]
	TNF-α	Lung cancer	Not shown	[143]
		Oral cancer	Not shown	[144]

^1^ Epidermal growth factor; ^2^ Glycoprotein nonmetastatic B; ^3^ Pleiotrophin; ^4^ Inslin-like growth factor; ^5^ Platelet-derived growth factor-BB; ^6^ Matrix metalloproteinase 9; ^7^ High mobility group box 1; ^8^ Milk-fat globule-epidermal growth factor-VIII.

### 3.1. CC and CXC Chemokines Are Secreted by TAMs to Maintain Tumor Cell Stemness Characteristics

Notably, the current study shows that TAMs maintain the characteristics of CSCs by secreting CC chemokines, such as CCL2, CCL5, CCL7, CCL8, and CCL17 (Table 3) (Figure 1). As described in Section 2.2.1, β-catenin secretion by BCSCs directly regulates CCL2 expression, promoting macrophage recruitment and TAM polarization. TAMs also express CCL2 and promote the maintenance of BCSC properties [48]. Similarly, CCL5, a chemokine that induces the recruitment and activation of macrophages, has also been found to be secreted by TAMs to promote CSC properties. In GBM, TAMs secrete CCL5, which enhances GSC stemness and tumor invasion via the CCL5–CCR5 pathway [52]. CCL5 activates β-catenin/STAT3 signaling to promote the self-renewal and tumor metastasis of prostate CSCs (PCSCs), and its knockdown significantly inhibits the metastasis of prostate cancer and the self-renewal capacity of PCSCs [110]. TAMs secrete CCL7, which binds to CCR3 to activate MMP9 expression in ovarian cancer cells, significantly increasing their invasiveness [111]. In addition, TAMs secrete large amounts of CCL8 in the TME, binding to receptors CCR1 and CCR5 on glioma cells, which activates ERK1/2 and can induce the aggressive and stemness characteristics of GBM [112]. However, TAM-derived CCL-17, CCL-22, TGF-β, IL-10, arginase 1, and galectin-3 can significantly reduce tumor growth and metastatic sites, but the specific mechanism remains unclear [145]. In summary, TAMs can secrete CC chemokines to support the growth of CSCs and targeting these chemokines may effectively block the positive feedback loop-like association between CSCs and TAMs.

TAMs also secrete CXC chemokines (CXCL1 and CXCL7) that support CSC-like characteristics (Table 3). M2 TAMs secrete CXCL1 in breast cancer and promote tumor metastasis. The use of CXCL1 promoter inhibitors reduced the migration of breast cancer cells and the number of BCSCs, suggesting that TAMs may support BCSC survival by secreting CXCL1 [116]. In addition, CXCL7 secretion was significantly positively correlated with higher numbers of TAMs and the enhanced stemness of GBM. FGL2 produced by GBM induces macrophage recruitment and promotes macrophages to exhibit TAM-like properties. Activated TAMs secrete CXCL7 via the CD16/SYK/PI3K/HIF1α pathway, thereby enhancing the stem-like characteristics of GBM cells [73]. In conclusion, TAMs secrete CC and CXC chemokines to maintain CSC stemness. Therefore, blocking the secretion of these chemokines can block CSC–TAM interactions, thus effectively inhibiting tumor growth and metastasis.

### 3.2. TAMs Support the Stemness of Tumor Cells by Secreting ILs

TAMs can also promote the self-renewal and metastasis of CSCs by secreting ILs. IL-6, a common IL factor, plays a vital role in promoting tumorigenesis. Activation of the TLR4 signaling pathway on microglia promotes the secretion of IL-6, and IL-6 can support GSC in regulating tumor growth [89]. In human HCC samples, the same results were observed. TAMs activated STAT3 to produce IL-6 and induced an increase in the number of CD44^+^ hepatoma cells [119]. Another study reported that M2 TAMs could also secrete IL-6 through the WNT ligand WNT5B, inducing the enrichment of CSCs in ovarian cancer [62]. Moreover, CSC-derived WNT ligands can drive the activation of M2 macrophages. Thus, a positive feedback loop is formed between CSCs and M2 TAMs through WNTs. In addition, M1 macrophages can secrete IL-6. In oral squamous cell carcinoma cells, M1 TAMs can promote EMT and induce the CSC phenotype by upregulating the expression of MME and MMP14. However, M1 TAMs secrete IL-6, increasing CSC stemness by activating the JAK/STAT3 pathway [141]. These findings indicate that TAMs secrete IL-6 through the TLR4 signaling pathway and STAT3 pathway and that IL-6 promotes the chemoresistance and invasiveness of CSCs by activating the JAK/STAT3 pathway and WNT pathway in CSCs, forming a CSC–TAM–CSC positive feedback loop.

TAMs can also secrete IL-10, IL-8, IL-12, and IL-35, which can promote the stemness characteristics of tumor cells (Figure 1). In NSCLC, IL-10 is derived from TAMs via the JAK1/STAT1/NF-κB/Notch1 signaling pathway and induces CSC-like properties in NSCLC cells. Blocking IL-10/JAK1 signaling significantly inhibits TAM-mediated CSC-related genes, such as Sox2, Oct4, and Nanog [121]. This pathway may provide a new potential therapeutic target for the treatment of NSCLC. In addition, IL-8 secreted by TAMs in ovarian cancer via the STAT3 pathway can also increase the stemness characteristics of OCSC [146]. Notably, in GBM, GSCs express higher amounts of active spin cleavage-like products (ALPs) than non-GSCs. ALPs promote secretion of IL-12 by M1 TAMs, which can enhance the activity of GSCs and drive the formation of the GSC niche [142]. In addition, the expression of RON signaling in TAMs induces the secretion of IL-35, which supports the self-renewal and metastasis of CSCs; however, the specific mechanism remains under investigation [123]. Together, these findings reveal that TAMs secrete ILs to promote the expression of CSC genes in various cancer models. In addition, M1 macrophages can secrete IL-6 and IL-12, inducing the expression of CSC genes. Studies continue to find more IL factors secreted by TAMs, and they play a crucial role in the maintenance of CSC stemness, but further research is required to clarify the precise role of TAMs in the maintenance of CSC stemness.

### 3.3. Other Cytokines and Soluble Protein Molecules Secreted by TAMs to Promote the Stemness of Tumor Cells

TAMs can also directly support tumor stemness by secreting cytokines, such as TGF-β1, FGF, and PGE2 (Figure 1). TAMs secrete more TGF-β1 than macrophages of other phenotypes, and TGF-β1 activates EMT and acquires CSC-like properties [124]. Furthermore, FGF expression in macrophages significantly increases the stemness of GBM cells. FGF1, the most abundant FGF in the FGF family, was upregulated in TAMs cocultured with GBM and enhanced the stemness characteristics of GMB [147]. In addition, in a breast cancer model, TAMs were noted to establish a paracrine EGFR/STAT3/Sox2 signaling pathway and increase Sox2, Oct4, Nanog, AbcG2, and Sca-1 gene expression [130]. TAMs can also secrete another cytokine, PGE2. Overexpression of IL-33 by colon cancer cells induces macrophage recruitment and stimulates them to produce PGE2, thereby supporting the stemness of colon cancer cells [148]. These findings show that TAMs are crucial in supporting CSCs and targeting the cytokines that facilitate this process can block tumor cells from acquiring or maintaining stemness characteristics.

TAMs can also support CSCs by secreting soluble protein molecules, such as GPNMB, TNF-α, pleiotrophin (PTN), growth factor-BB, immunomodulatory cationic antimicrobial peptide 18/LL-37 (hCAP-18/LL-37), and S100A9. GPNMB is a glycoprotein that is highly expressed in macrophages and microglia and promotes inflammation. However, in mouse tumor models, M2 TAMs preferentially express soluble GPNMB and bind to the CD44 receptor on tumor cells to trigger CSC proliferation [131]. In addition, in clear-cell renal cell carcinoma (ccRCC), TAMs secrete TNF-α, which may upregulate CD44 expression through NF-κB signaling and enhance CSC stemness [128]. PTN is also a soluble protein molecule secreted by TAMs, which is significantly positively correlated with the expression of PTPRZ1 (PTN receptor) in lymphoma. PTN induces the generation of lymphoma stem cells via the β-catenin pathway [132]. In addition, GSCs can upregulate the expression of PTPRZ1 and activate PTPRZ1 signaling by binding to PTN secreted by M2 TAMs to maintain GSC stemness [133]. Together, these results indicate that PTN secreted by TAMs plays a crucial role in the maintenance of CSC stemness. A recent study revealed that TAMs can also secrete IGF-1 and IGF-2 to promote PI3K/AKT/mTOR signaling and increase the stemness characteristics of thyroid cancer [134]. In pancreatic ductal adenocarcinoma, TAMs secrete hCAP-18/LL-37, creating a paracrine niche beneficial to CSCs [136]. S100A9, a secreted protein associated with the TME, is significantly upregulated in TAMs [149]. It activates the NF-κB signaling pathway in a Ca2^+^-dependent manner to enhance the stem cell-like properties of hepatoma cells. Simultaneously, treatment of tumor cells with S100A9 significantly increased CCL2 expression, recruited and polarized more macrophages, and formed a CSC–TAM positive feedback loop [137]. These findings suggest that TAMs produce abundant protein molecules in the TME, and these abundant proteins promote the generation and maintenance of CSCs by activating CSC-related pathways.

### 3.4. TAMs Promote Tumor Cell Stemness through Surface Protein Molecules

TAMs can also directly bind to CSCs to support CSC stemness by expressing corresponding protein molecules on the cell surface, including CD90, EphA4, and SIRP1α. In breast cancer, EMT programmatically upregulates the expression of CD90 and EphA4. CD90 directly binds to receptors on TAMs to mediate physical effects and activate the NF-κB pathway in CSCs and induce the production of cytokines to maintain stemness [21] (Figure 1). In addition, CD47 expression was observed to be elevated in CD133^+^ lung cancer cells. CD47 also binds to SIRP1α on macrophages, thus inhibiting phagocytosis [20] and supporting immune escape. In conclusion, the surface protein molecules expressed on the surface of TAM-CSC, after sufficient contact, play a significant role in immunosuppression; these surface molecules can be used as immune checkpoints to improve the efficacy of immunotherapy.

### 3.5. TAMs Promote Tumor Cell Stemness by Secreting Extracellular Vesicles

TAM-derived extracellular vesicles can also promote tumor invasion. The expression of microRNA-21-5p is upregulated in M2 macrophage-derived extracellular vesicles and combines with KLF3 to induce Pa-CSC differentiation [140]. Accordingly, nanocarriers inspired by M2 macrophage microvesicles can penetrate tumor tissue to improve the accessibility of CSCs [150]. Therefore, the use of extracellular vesicles can produce an effective tumor treatment effect.

## 4. Therapeutic Strategies Targeting CSCs and TAMs

**Figure 2:**
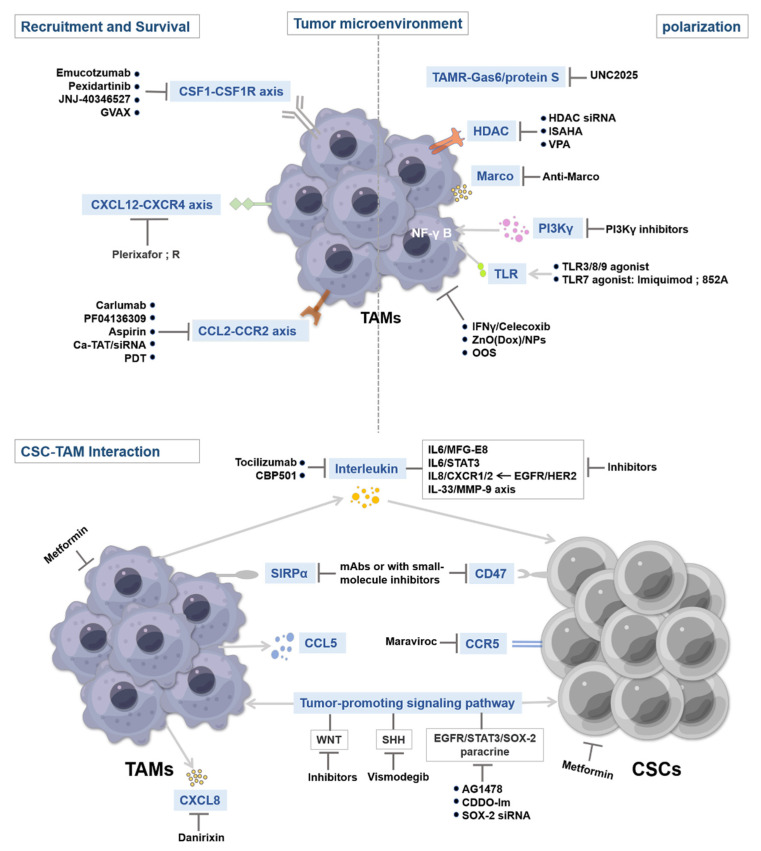
Targeting strategies of CSCs and TAMs in cancer therapy. Key pathways regulating CSC and TAM properties during tumorigenesis and progression have been identified, including TAM survival, recruitment, polarization, and phagocytosis and maintenance of CSC stemness. Targeting these pathways can help regulate CSC properties and TAMs and interfere with the interaction between them, thereby inhibiting the development of CSCs and providing more effective therapeutic strategies.

Macrophages constitute the bulk of immune cells. They have tumor-promoting and immunosuppressive roles in cancer [151,152]. For example, the number of TAMs in human tumors is positively correlated with tumor grade and negatively correlated with patient survival in cancers such as GBM, lymphoma, breast, and pancreatic cancers [153,154,155,156,157]. However, when TAMs lose immunosuppressive signals or undergo repolarization, survival is increased in mice and in patients with different types of cancer [158]. In recent tumor treatment studies, macrophage therapy has received great attention due to the contribution of macrophages to CSC growth, metastasis, and promotion of the CSC niche. Several common immunotherapeutic strategies include depleting monocytes to prevent TAM production and recruitment, inducing macrophage polarization toward the M1 phenotype and promoting phagocytosis, targeting TAMs supporting the CSC niche, and suppressing CSC occurrence and metastasis [159]. These strategies are now being tested to enhance antitumor immunity, in conjunction with either conventional chemo-radiotherapy or T cell–mediated immunotherapy [159]. We discuss some potential pathways that can translate into therapeutic strategies that interfere with the interaction between TAMs and CSCs, inhibiting TAM-mediated immunosuppression. Figure 2 presents several key targets that regulate CSC and TAM properties during tumor progression. In addition, we also list the relevant agents targeting TAM in ongoing clinical trials (Table 4).

### 4.1. Targeting Tumor-Associated Macrophage Recruitment and Survival

TAMs mainly originate from monocyte precursor cells in the circulation system. One strategy to deplete TAMs is to block the replenishment of TAMs through monocyte recruitment or by targeting M2 macrophage markers to specifically clear TAMs from the TME (Figure 2).

#### 4.1.1. CSF1–CSF1R Inhibitors

Blocking the CSF1–CSF1R signaling pathway can inhibit macrophage recruitment to the CSC niche (Figure 2). The CSF1–CSF1R axis can promote TAM differentiation, survival, and recruitment [160]. Blockade of this signaling pathway can greatly reduce the number of TAMs or repolarize M2 macrophages to M1 macrophages in the TME [161,162,163]. Studies have reported that blockade of the CSF1–CSF1R axis improved the effects of immunotherapy [164,165]. Emactuzumab, a monoclonal antibody to CSF1, blocks the activation of this signaling pathway by inhibiting CSF1–CSF1R dimerization. Emactuzumab treatment can reduce the number of F4/80^+^ TAMs in CSC niches and increase the ratio of CD8^+^/CD4^+^ T cells, thereby improving antitumor immune function. Emactuzumab combined with paclitaxel can significantly reduce the number of CSF1R^+^ TAMs and effectively inhibit the development of advanced solid tumor CSCs [166]. Pexidartinib, an inhibitor of tyrosine kinases on CSF1R, can inhibit the activation of TAMs to block their tumor-promoting effects, thereby reducing the CSC count in the TME. Pexidartinib has been approved by the FDA as an alternative to surgery for tenosynovial giant cell tumor [167,168,169]. The use of monoclonal antibodies or small-molecule inhibitors that target the CSF1–CSF1R signaling pathway can successfully reduce the number of TAMs in the CSC niche and increase the number of infiltrating T cells, thereby enhancing antitumor immunity (Figure 2) [160].

In addition, the granulocyte-macrophage colony-stimulating factor (GM-CSF) gene-transduced vaccine (GVAX), a cell-based tumor vaccine transduced by the GM-CSF gene, can be used to block the CSF2–CSF2R signaling pathway, which inhibits TAM activation [170]. Following GVAX inoculation, sustained local release of GM-CSF significantly increased levels of M1 macrophages, dendritic cell populations, and CD8^+^ T cells, exerting antitumor immunity effects [170]. In addition, the IL-2 vaccine can also induce specific antitumor immunity in mice, and the combination of GVAX and IL-2 vaccine was noted to have better antitumor effects than the single treatment [171]. These results suggest that GVAX can induce strong tumor immunity and can be used in tumor immunotherapy.

#### 4.1.2. CCL2–CCR2 Inhibitors

The migration and recruitment of monocytes from bone marrow to the CSC niche occurs mainly via the CCL2–CCR2 signaling pathway. Therefore, targeting the CCL2–CCR2 axis effectively inhibits the number of invasive TAMs in the CSC niche [172,173] and synergistically improves the efficacy of chemo-radiotherapy and immunotherapy (Figure 2) [174]. TAM activity can also be inhibited by monoclonal antibodies or small-molecule inhibitors targeting CCL2 or CCR2. Carlumab and PF04136309 play a key role in targeting the CCL2–CCR2 signaling pathway. Carlumab is a monoclonal antibody with a high binding ability to CCL2, which can reduce the number of CD68^+^ TAMs in the CSC niche, increase the phagocytic ability of macrophages, and effectively control the occurrence and metastasis of CSCs [175,176]. PF04136309, a CCR2 antagonist, inhibits the metastatic infiltration of CCR2^+^ macrophages and reduces the proportion of TAMs in the CSC niche [177]. In addition, in esophageal cancer cells, aspirin can reduce the recruitment of M2 macrophages, the expression of CSC marker genes CD90 and Nanog, and the ability of CSCs to form spheroids by inhibiting CCL2 expression on TAMs [178].

The combined application of siRNA and drugs to block the CCL2/CCR2–STAT3 axis can inhibit the activity of TAMs in the CSC niche, thus enhancing the phagocytic activity of macrophages and inhibiting tumor growth and metastasis [179]. In addition, siRNA complexed with TAT cell-penetrating peptide (Ca-TAT) can silence genes more effectively than traditional antibody neutralization [75].

Low-dose photodynamic therapy can significantly inhibit CCL2 secretion in MSCs, possibly limiting the infiltration of precancerous macrophages [180]. However, whether it can inhibit CSC secretion of CCL2 remains to be further investigated.

#### 4.1.3. CXCL12–CXCR4 Inhibitors

The CXCL12–CXCR4 signaling pathway is associated with macrophage recruitment, and its blockade in combination with traditional therapy has shown antitumor efficacy (Figure 2) [181]. Some small-molecule antagonists against the CXCL12–CXCR4 axis can be used for antitumor therapy, such as small peptide CXCR4 antagonists, nonpeptide CXCR4 antagonists, anti-CXCR4 antibodies, modified CXCL12 agonists, and antagonists [182,183]. Plerixafor is a CXCR4 antagonist that inhibits CXCL12–CXCR4-mediated chemotaxis and reduces macrophage recruitment in CSC niches. CXCR4 is highly expressed in most CSCs, and the combination of nonpeptide CXCR4 antagonists with etoposide and cisplatin reduces CSC proliferation and inhibits tumor growth [184]. In addition, highly diffused CD133^+^ CXCR4^+^ cells (metastasis promoter cells, MICs) can trigger distant metastasis and resist conventional chemotherapy. A new CXCR4 inhibitory peptide can prevent the expression of CD73, CD38, and IL-10 by MICs, thus inhibiting macrophage transformation into TAMs and rescuing the antitumor cytotoxic effects of T cells [185].

However, these target molecules for TAM-targeted therapy may exist in cell populations other than TAMs. For example, CCR2 and CXCR4 are also expressed on lymphocytes [186,187,188]. Simultaneous targeting of these molecules may lead to altered functions of these immune cells as well as various other complications; therefore, future studies should attempt to identify more specific targets.

### 4.2. Targeting TAM Activation

Another strategy is to reprogram macrophages to repolarize toward the M1 antitumor phenotype from the M2 tumor-promoting phenotype and enhance macrophage phagocytosis to produce antitumor effects (Figure 2).

#### 4.2.1. TAMR Inhibitors

TAMR, a collective term for Tyro3, Axl, and MerTK, is a group of tyrosine kinase receptors that are ubiquitously expressed on CSCs and various immune cells. Axl and MERTK are overexpressed in CSCs, which can promote M2 polarization of macrophages [189,190,191]. The interaction of TAMR with ligands Gas6 and protein S can stimulate M2 polarization, promote the production of immunosuppressive cytokines, prevent the activation of immune T cells, limit the inflammatory response, and favor CSC survival [192]. Therefore, blocking the TAMR signaling pathway may effectively inhibit the activities of TAMs and CSCs (Figure 2). Researchers have discovered that UNC2025, a small-molecule inhibitor of MERTK, can inhibit macrophage transformation into TAMs, increase the phagocytosis of M1 macrophages, and reduce the CSC count [193].

#### 4.2.2. Anti-MARCO Antibody

MARCO, also known as SCARA2, is a macrophage receptor with a collagenous structure that drives the transformation of macrophages into TAMs. Macrophages expressing MARCO represent a subset of macrophages with a protumor and anti-inflammatory immunosuppressive phenotype [194,195]. Therefore, targeting MARCO may effectively inhibit TAM activation (Figure 2). In breast and colon cancer and melanoma models, the use of anti-MARCO antibodies can reprogram TAMs toward the M1 phenotype, reduce the proportion of TAMs in the CSC niche, increase tumor immunogenicity, and improve the efficacy of antitumor immunotherapy [194,196].

#### 4.2.3. Toll-Like Receptors and Stimulator of Interferon Gene Agonists

Toll-like receptors (TLRs) on macrophages can trigger TAM repolarization. TLRs can bind to some endogenous molecules, participate in antivirus and antitumor regulation by activating NF-κB, and produce various immune-stimulating cytokines, including type I IFN, which increase the expression of MHC-I, promote the repolarization of TAMs to the M1 phenotype, and enhance the specific recognition of CSCs by the immune system [197,198]. Encouraging results have been obtained within the TLR3, TLR7, TLR8, and TLR9 agonists in mouse tumor models. Among these, the TLR7 agonists imiquimod and 852A have shown potential antitumor ability and durable therapeutic effects in preclinical trials [199]. Another key factor regulating innate immunity is the stimulator of interferon genes (STING), which also leads to the production of immune-stimulating cytokines [200]. Activation of TLRs and STING can induce good antitumor effects in the body [201,202,203]. On the basis of these results, TLR and STING agonists are being used in CSC treatment trials in combination with other chemotherapy and immunotherapies (Figure 2) [202,203].

#### 4.2.4. Others

PI3Kγ signaling can promote the immunosuppressive activity of TAMs. In melanoma, lung, and pancreatic cancer cell models, the blockade of PI3Kγ expression in TAMs activates NF-κB, which promotes the immunostimulatory transcriptional program, rescues CD8^+^ T cell cytotoxicity, and promotes the regression of CSCs and prolongs survival in mouse models in combination with immune checkpoint inhibitor therapy [151,158].

TAMs can express histone deacetylases (HDACs), and HDAC inhibitors can perform epigenetic reprogramming of macrophages, repolarizing them toward the M1 phenotype, and induce T cell toxicity [204]. Inhibition of HDAC2 expression in TAMs through siRNA knockdown or by using pharmacological inhibitors (ISAHA and VPA) can induce TAM repolarization, activate the antitumor phenotype of macrophages, help T-lymphocyte responses, and increase the sensitivity of CSCs to chemotherapy and immune checkpoint inhibition [204]. Furthermore, the histone methyltransferase EZH2 utilizes a methylation modification to inhibit miR-454-3p and promotes the m6A modification of PTEN to polarize macrophages toward the M2 phenotype. Of note, combined treatment with EZH2 inhibitors and PD-1 inhibitors restored the cytotoxicity of macrophages and blocked the progression of CSCs [205,206,207,208,209,210,211,212].

IFN-γ is a potent antiviral bioactive substance, and celecoxib is a nonsteroidal anti-inflammatory drug. In a study, their combination decreased the M2/M1 macrophage ratio in the CSC niche reduced the expression of MMP-2, MMP-9, and VEGF of TAMs; and inhibited microangiogenesis in the CSC niche [213]. Thus, IFN-γ and celecoxib may inhibit CSC development by regulating the ratio of M2/M1 macrophages in the TME.

Folic acid oral solution (OOS) has anti-inflammatory, antioxidant, and antitumor effects and can inhibit M2 macrophages in vitro and in vivo without side effects. In addition, OOS can target plastic CSCs as well as the TME that supports CSCs [214]. Therefore, OOS, which can counteract the hypoxic state and inhibit the activity of TAMs and CSCs in the TME, can be a candidate for adjuvant therapy in malignant tumors.

ZnO/NPs loaded with the chemotherapeutic drug doxorubicin (Dox) are multifunctional and multitargeted nanodrug carriers. ZnO(Dox)/NPs can promote Dox-induced polarization of macrophages to an M1 phenotype in vivo and increase the sensitivity of CSCs to doxorubicin treatment. ZnO(Dox)/NPs can also effectively downregulate CD44, a key surface marker of CSCs, reduce CSC stemness, and inhibit CSC development [215].

### 4.3. Targeting CSC–TAM Interaction

The molecules that mediate CSC–TAM interaction are important for maintaining CSC stemness. Accordingly, specifically interfering with this interaction and disrupting the plasticity of CSCs and TAMs in the TME may lead to more effective antitumor responses (Figure 2) [19,216].

#### 4.3.1. Blockade of the SIRP-α–CD47 Pathway

SIRP-α can inhibit the phagocytosis of CSCs by TAMs and promote tumor immune evasion (Figure 2) [217]. Therefore, blocking the CD47–SIRP-α pathway can interfere with CSC–TAM interaction [218,219]. Several studies are currently underway to test the efficacy of monoclonal antibodies and small-molecule inhibitors targeting SIRP-α or CD47.

CD47 deletion may reduce the stemness properties of glioma CSCs. Anti-CD47 antibody therapy increased the phagocytic activity of macrophages to glioma stem cells, decreased cancer stemness, and resulted in a significant inhibition of tumor growth [219]. Subsequently, the combination of CD47 blockers and the antimicrotubule drug cabazitaxel promoted programmed cell clearance (PRCR) in TNBC cells as well as the activation of NF-κB and generation of immune-stimulating cell factors, which promoted macrophage polarization to the M1 phenotype and inhibited the activity and self-renewal of CSCs [220]. Accumulating evidence suggests that targeting CD47 may be a safe, effective, and promising treatment strategy for CSCs. Similarly, anti-SIRP-α antibodies can increase macrophage-mediated phagocytosis and T-cell-mediated cytotoxicity to CSCs and reduce the resistance of CSCs to antiangiogenic therapy [221,222]. Notably, blocking the SIRP-α-CD47 signaling pathway in combination with chemoradiotherapy can further improve the antitumor therapeutic effect [19]. Moreover, targeting E3-ligase-mediated CD47 degradation to boost anticancer immunity also has been discussed [223]. The above findings suggest that blocking the SIRP-α-CD47 signaling pathway with monoclonal antibodies or small-molecule inhibitors can effectively block CSC–TAM interaction, thereby inhibiting the progression of CSCs and reducing the tumor burden in vivo.

#### 4.3.2. Targeting IL-Mediated Signaling Pathways

Cytokines such as IL-6 and IL-10 are essential for maintaining CSC stemness and promoting TAM activation, and their inhibitors have shown potent antitumor activity [224]. IL-6 secreted by TAMs can promote the proliferation and development of CSCs, and IL-6 levels correlate with the specific phenotype of CSCs. CD44^+^ HCC cell lines exhibit CSC activity in vitro; however, tocilizumab, a monoclonal antibody to the IL-6 receptor, can inhibit IL-6 function and downregulate TAM-mediated CD44^+^ cell activity, reducing the stemness of CD44^+^ cells [119]. TAMs can activate the Sonic Hedgehog (SHH) and STAT3 signaling pathways in CSCs by releasing milk fat globule-EGF factor 8 (MFG-E8), which plays a vital role in regulating the activity of CSCs and enhances the resistance to antitumor drugs [225,226]. IL-6 can increase the tumorigenic activity of CSCs by enhancing MFG-E8-induced activity, and the combined blockade of IL-6 and MFG-E8 can significantly reduce the number of primary tumor-derived CSCs, whereas blockade of IL-6 alone or MFG-E8 has only a partial antitumor effect. Furthermore, the inhibition of the MFG-E8-activated SHH and STAT3 pathways significantly increased cisplatin (CDDP)-induced apoptosis of CSCs [139]. In addition, blocking the IL-6/STAT3 signaling pathway has also shown some antitumor activity, with STAT3 involved in the recruitment and polarization of TAMs [64,227]. Anti-IL-6 or anti-IL-6R antibodies or small-molecule inhibitors targeting STAT3 can inhibit the activity of CSCs and M2 polarization of macrophages, thus inhibiting the progression of CSCs [39,91]. STAT3 knockdown by siRNA prevented tumor cell proliferation and blocked CSC-mediated immunosuppression [228,229].

A study reported an antitumor drug candidate, CBP501, which inhibits the lipopolysaccharide-induced secretion of TNF-α, IL-6, and IL-10 from TAMs. Coculture of CBP501 with lipopolysaccharide-treated TAMs inhibited the formation of tumor spheroid cells. In addition, CBP501 can also inhibit the expression of CSC marker ABCG2 by inhibiting the interaction between VCAM-1^+^ cancer cells and VLA-4^+^ macrophages [143].

IL-8 and CXCR1/2 are a pair of homologous receptors. IL-8 enhances the tumor-promoting activity of TAMs and promotes the progression of CSCs by helping the interaction between CSC and TAM. The IL-8/CXCR1/2 signaling pathway is partially mediated by the EGFR/HER2 signaling pathway. Therefore, the simultaneous application of CXCR1/2 inhibitors and HER2 inhibitors in cancer therapy can inhibit the tumor-promoting activity of TAMs, inhibit the proliferation and migration of CSCs, and improve the overall survival rate of patients with cancer [230,231].

IL-33 can upregulate the expression of MMP-9 in TAMs. MMP-9 can modify activated receptors on NK cells, CD8^+^ T cells, CD4^+^ T cells, as well as inhibit the expression of antigen-presenting molecule MHC-I on the surface of CSCs, which interferes with the interdependence between CSCs and TAMs [232]. These findings suggest that IL-33-induced TAMs can significantly reduce the toxic effects of immune cells on CSCs through MMP-9 blockade [233,234]. Therefore, the researchers reasoned that the antitumor effect of immune cells could be enhanced by inhibiting the proteolytic activity of MMPs, namely by targeting the IL-33–TAMs–MMP-9 axis-mediated immune tolerance of CSCs; this represents a new therapeutic modality against CSCs.

#### 4.3.3. Others

Targeting promoting-cancer pathways, such as WNT, Notch, and Hedgehog, can help disrupt the interdependence between CSCs and TAMs [235]. During CSC–TAM interaction, activated WNT signaling initiated by TAMs can promote the chemoresistance and invasive phenotypes of CSCs [62], suggesting that targeting the WNT pathway may inhibit the activity of CSCs and TAMs.

The EGFR/STAT3/Sox2 paracrine signaling pathway, which mediates CSC–TAM interaction, can promote the expression of the CSC marker gene Sox2, thus enhancing CSC stemness, and increase the ability of drug excretion and chemotherapy resistance in vivo [130]. Therefore, knockdown of Sox2 in CSCs by siRNA can inhibit the TAM-mediated stemness of CSCs, thereby inhibiting tumor progression [130]. In addition, targeting EGFR and STAT3 by small-molecule inhibitors AG1478 and CDDO-Im, respectively, can also effectively block this signaling pathway [130]. Other pathway inhibitors, such as Vismodegib, an SHH pathway inhibitor, can be used to treat metastatic or advanced localized basal cell carcinoma [236]. In addition, imatinib is a tyrosine kinase inhibitor that can inhibit STAT6 phosphorylation and nuclear translocation, thereby promoting macrophage-induced phagocytosis and reducing CSC growth and migration [237]. Notably, these signaling pathways also act on normal tissue cells. Therefore, to minimize adverse effects, researchers should find therapeutic pathways that specifically target CSCs or TAMs, which is undoubtedly a major challenge.

One study demonstrated that inhibition of CSC-specific POSTN and its related pathways or the GBM derivative CCL5 disrupted CSC–TAM interaction in mouse models of GBM and prostate cancer [69,110]. Maraviroc is a CCR5 antagonist that significantly inhibits the proliferation, colony formation, and migration of CCR5^+^ CSCs [52].

Chemokine ligand 8 (CXCL8) is a key chemokine secreted by TAMs that can mediate CSC–TAM interaction. CXCL8 significantly increased the migration, invasion, and EMT events of breast cancer cells, as well as the self-renewal of BCSCs [117]. Although danirixin is a selective antagonist of CXC chemokine receptor 2 (CXCR2), in vivo analysis confirmed that it can also inhibit the activity of CXCL8, thus inhibiting the self-renewal and metastasis properties of BCSCs [238,239]. These results suggest that danirixin treatment significantly abrogates TAMs/CXCL8-mediated tumor-promoting efficacy.

Metformin is a traditional drug commonly used to treat type 2 diabetes. It can significantly reduce the proportion of ALDH^+^CD133^+^ CSCs in patients with ovarian cancer and can decrease HCC stemness [240,241]. In addition, metformin may also increase macrophage phagocytosis by reprogramming the functional activity of macrophages [242], thereby affecting the interaction of TAMs with CSCs, reducing CSC levels, and inhibiting tumor growth.

Survivin, an antiapoptotic protein, is present in human adipose tissue-derived stem cells (HASCs) and is a diagnostic marker of tumor recurrence. One study reported that HASCs isolated from obese patients had significantly elevated survivin expression; at the same time, the antiapoptotic capacity of HASCs and the activity of soluble proinflammatory cytokines (such as IL-1β) secreted by M1 macrophages were also improved [243]. Therefore, targeting survivin may present a new approach to cancer treatment.

Despite the attractive therapeutic strategies targeting CSCs, they lack efficient and specific CSC targets. Furthermore, due to their high plasticity, CSCs may become reestablished after the treatment-induced loss of stemness. Similarly, some TAM-targeting strategies, such as antagonizing CSF1R, also have only a weak antitumor effect, which may be due to some altered pathways in the TME leading to unknown therapy resistance mechanisms of TAMs.

## 5. Summary and Perspective

CSCs represent a small portion of the cancer cells in a tumor, but they have very powerful self-renewal properties, are often resistant to chemotherapy and radiation therapy, and promote tumor recurrence and metastasis. This article reviewed the molecular mechanism of how CSC–TAM interaction maintains tumor progression. This bidirectional effect is manifested at several levels, including the role of CSCs in promoting the recruitment and polarization of TAMs and the role of TAMs in promoting CSC stemness and CSC niche formation. We also described potential therapeutic strategies, including various monoclonal antibodies, small-molecule inhibitors, agonists, and antagonists, that involve targeting (1) tumor-associated macrophage recruitment and survival, (2) tumor-associated macrophage activation, and (3) cytokines and chemokines involved in CSC–TAM interaction. However, most treatments are still in preclinical studies. A major challenge is the high plasticity of CSCs and the lack of efficient and specific therapeutic targets. In addition, macrophages play various important roles in the immune system, and excessive depletion of these cells can seriously affect immune function. To address these issues, new therapeutic strategies are required to target markers of TAMs and CSCs efficiently and specifically to precisely target these tumor immunosuppressive cell populations. Furthermore, current knowledge of TAMs and CSCs relies heavily on tumor engraftment analysis in syngeneic and xenograft models; these models do not exhibit the exact same TME as the primary tumor, nor do they fully mimic the interaction between human CSCs and TAMs. Therefore, developing a mouse model with a human-like immune system that can support the growth of human primary tumors will elucidate the relationship between TAMs and CSCs.

In conclusion, the interaction between TAMs and CSCs during tumor progression constitutes a positive feedback loop and exerts structural maintenance and protective effects on the CSC niche. Although many therapeutic strategies have been proposed to target TAMs and CSCs, the results remain unsatisfactory. Further elucidation of the mechanisms and roles of CSC–TAM interaction in the progression of CSCs and using TAM-specific and CSC-specific targeted immunotherapy to treat CSCs are warranted to develop more effective therapies with minimal adverse effects.

## Figures and Tables

**Table 4:** Drugs targeting the TAM-related pathways in clinical trials.

Targets	Drugs	Cancer Type	Trial Phase	ClinicalTrials.Gov ID
CSF-1/CSF-1R	CM082	Small cell lung cancer	II	NCT03904719
	ABSK021	Advanced solid tumor	I	NCT04192344
	AMB-051	Tenosynovial giant cell tumor	II	NCT04731675
	SNDX-6532	Unresectable intrahepatic cholangiocarcinoma	II	NCT04301778
	Chiauranib	Triple-negative breast cancer	II	NCT05336721
		Small cell lung cancer	III	NCT04830813
	NMS-03592088	Acute myeloid leukemia Chronic myelomonocytic leukemia	I/II	NCT03922100
	Pexidartinib	Tenosynovial giant cell tumor	III	NCT04488822
	Q702	Advanced solid cancer	I	NCT04648254
	Surufatinib	Neuroendocrine tumor Non-hematologic malignancy	I/II	NCT05077384
		Hepatocellular carcinoma	II	NCT05171439
		Refractory metastatic digestive system carcinoma Primary peritoneal cancer	II	NCT05030246
	TPX-0022	Advanced metastatic solid tumor	I/II	NCT03993873
	Vorolanib	Refractory thoracic tumor	I/II	NCT03583086
		Metastatic renal cell cancer	III	NCT03095040
CCL2/CCR2	BMS-813160	Locally advanced pancreatic ductal Adenocarcinoma Pancreatic ductal adenocarcinoma	I/II	NCT03767582
CCL5/CCR5	Maraviroc	Colorectal cancer metastatic	I	NCT04721301
	Leronlimab	Solid tumor	II	NCT04504942
	POL6326	Advanced breast cancer	I/II	NCT04826016
		Metastatic breast cancer	III	NCT03786094
	Motixafortide	Pancreatic cancer	II	NCT04543071
CD40/CD40L	ABBV-368	Advanced solid tumors	I	NCT04196283
		Triple-negative breast cancer Non small cell lung cancer Metastatic solid tumor	I	NCT03893955
	ABBV-927	Advanced solid tumor Triple-negative breast cancer Non-small cell lung cancer Metastatic solid tumor	I	NCT03893955
		Pancreatic cancer	II	NCT04807972
	CDX-1140	Pancreatic cancer	II	NCT04536077
		Solid tumor Diffuse large B-cell lymphoma Mantle cell lymphoma	I	NCT03329950
		Ovarian clear cell adenocarcinoma	II	NCT05231122
		Melanoma	I/II	NCT04364230
		Metastatic triple negative breast cancer	I	NCT05029999
		Malignant epithelial neoplasm	I	NCT04520711
	GEN-1042	Malignant solid tumor Non-small cell lung cancer Colorectal cancer Melanoma Head and Neck squamous cell carcinoma Pancreatic ductal adenocarcinoma	I/II	NCT04083599
	Mitazalimab	Metastatic pancreatic ductal adenocarcinoma	I/II	NCT04888312
	NG-350A	Epithelial tumor Metastatic cancer	I	NCT05165433
	Selicrelumab	Colorectal cancer	I/II	NCT03555149
		Triple-negative breast cancer	I/II	NCT03424005
		Pancreatic adenocarcinoma	I/II	NCT03193190
	YH-003	Advanced solid tumor	I/II	NCT04481009
		Melanoma Pancreatic ductal adenocarcinoma	II	NCT05031494
	SL-172154	Ovarian cancer Fallopian tube cancer Primary peritoneal carcinoma	I	NCT04406623
		Cutaneous squamous cell carcinoma Head and Neck squamous cell carcinoma	I	NCT04502888
		Acute myeloid leukemia	I	NCT05275439
	BDB-001	Solid tumor	II	NCT04819373
		Pancreatic cancer Virus-associated tumor Non-small cell lung cancer Melanoma Bladder cancer Triple-negative breast cancer	II	NCT03915678
	BDC-1001	HER2 positive solid tumor	I/II	NCT04278144
	BNT-411	Extensive-stage small cell lung cancer	I/II	NCT04101357
	DSP-0509	Neoplasm	I/II	NCT03416335
	Imiquimod	Metastatic melanoma	I	NCT03276832
		Oral cancer	I	NCT04883645
		Squamous cell carcinoma	I	NCT03370406
	Resiquimod	Locally advanced solid tumor Metastatic solid tumor	I/II	NCT04799054
		Anaplastic astrocytoma	II	NCT01204684
	SBT-6050	HER2 positive solid tumor	I	NCT04460456
TLR9	AST008	Advanced or Metastatic merkel cell carcinoma	I/II	NCT03684785
	CMP-001	Head and Neck squamous cell carcinoma	II	NCT04633278
		Metastatic melanoma	II	NCT04698187
		Merkel cell carcinoma	II	NCT04916002
		Malignant colorectal neoplasm	I	NCT03507699
		Advanced malignancy Non-small cell lung cancer Ovarian cancer Urothelial cancer Solid tumor	III	NCT05059522
	Tilsotolimod	Advanced solid tumor	I	NCT04196283
		Malignant melanoma	II	NCT04126876
	SD-101	Metastatic uveal melanoma in the Liver	I	NCT04935229
		Hepatocellular carcinoma Intrahepatic cholangiocarcinoma	I/II	NCT05220722
		B-Cell Non-Hodgkin lymphoma	I	NCT03410901
		Metastatic pancreatic adenocarcinoma	I	NCT04050085
TLR3	Poly-ICLC	Hepatocellular carcinoma	I	NCT05281926
		Glioblastoma multiforme	I/II	NCT03665545
	Rintatolimod	Hematopoietic and lymphoid cell neoplasm	I/II	NCT04379518
		Prostate adenocarcinoma	II	NCT03899987
PI3Kγ signal pathway	Copanlisib	Non-small cell lung cancer	I	NCT04895579
		Diffuse large B-cell lymphoma	II	NCT04433182
		Refractory/Recurrent primary central system lymphoma	I/II	NCT03581942
		Follicular lymphoma Endometrial cancer	II	NCT04750941
		Mantle cell lymphoma	I/II	NCT03877055
		Head and Neck squamous cell carcinoma Hepatocellular carcinoma	I	NCT03735628
	Duvelisib	Non-Hodgkin lymphoma	I	NCT05065866
		Unresectable melanoma	I/II	NCT04688658
		Recurrent diffuse large B-Cell lymphoma	I	NCT04890236
	Eganelisib	Bladder cancer Urothelial carcinoma	II	NCT03980041
		Non-small cell lung cancer Melanoma Head and Neck squamous cell cancer Triple-negative breast cancer Adrenocortical carcinoma Mesothelioma High-circulating myeloid-derived suppressor cells	I	NCT02637531
		Breast cancer Renal cell carcinoma	II	NCT03961698
	Gedatolisib	HER2-positive breast cancer Metastatic breast cancer	II	NCT03698383
		Triple-negative breast cancer	I/II	NCT03911973
		Squamous cell lung cancer Solid tumor Head and Neck cancer Pancreatic cancer	I	NCT03065062
	Tenalisib	Locally advanced breast cancer Metastatic breast cancer	II	NCT05021900
CD47/SIRPα pathway	ALX-148	Microsatellite stable metastatic colorectal cancer	II	NCT05167409
		Aggressive B-Cell Non-Hodgkin lymphoma	I/II	NCT05025800
		Head and Neck squamous cell carcinoma	II	NCT04675294
		Acute myeloid leukemia	I/II	NCT04755244
		Gastroesophageal junction adenocarcinoma	II/III	NCT05002127
		Non-Hodgkin lymphoma	I	NCT03013218
	AO-176	Multiple myeloma	I/II	NCT04445701
		Solid tumor	I/II	NCT03834948
	HX-009	Advanced solid tumor	II	NCT04886271
		Relapsed/Refractory lymphoma	I/II	NCT05189093
	IBI-188	Acute myeloid leukemia	I/II	NCT04485052
		Lung adenocarcinoma Osteosarcoma	I	NCT04861948
	IBI-322	Advanced malignant tumor Lymphoma	I	NCT04338659
		Non-small cell lung cancer	II	NCT05296278
		Hematologic malignancy	I	NCT04795128
		Advanced malignancy	I	NCT04328831
		Myeloid tumor	I	NCT05148442
	IMC-002	Advanced cancer	I	NCT05276310
		Solid tumor Lymphoma	I	NCT04306224
	IMM-01	Acute myeloid leukemia	I/II	NCT05140811
	IMM-0306	B-cell non-Hodgkin’s lymphoma	I	NCT04746131
	Lemzoparlimab	Acute myeloid leukemia Myelodysplastic syndrome	I	NCT04912063
		Multiple myeloma	I	NCT04895410
	Magrolimab	Malignant brain tumor	I	NCT05169944
		Recurrent neuroblastoma	I	NCT04751383
		Solid tumor	II	NCT04827576
		Recurrent acute myeloid leukemia	I/II	NCT04435691
		Metastatic colorectal cancer	II	NCT05330429
		Multiple myeloma	II	NCT04892446
		Head and neck squamous cell carcinoma	II	NCT04854499
		Metastatic colorectal cancer	II	NCT05330429
		Multiple myeloma	II	NCT04892446
		Urothelial carcinoma Bladder cancer	I/II	NCT03869190
	RRx-001	Central nervous system neoplasm	I	NCT04525014
		Small cell lung carcinoma	III	NCT03699956
		Small cell carcinoma Non-small cell lung carcinoma Neuroendocrine tumor Ovarian epithelial cancer	II	NCT02489903
	TTI-622	Multiple myeloma	I	NCT05139225
		Fallopian tube cancer Primary peritoneal carcinoma	I/II	NCT05261490
		Acute myeloid leukemia Diffuse large B-Cell lymphoma	I	NCT03530683
STING pathway	SNX-281	Advanced solid tumor Advanced lymphoma	I	NCT04609579
	BMS-986301	Advanced solid cancer	I	NCT03956680
	GSK-3745417	Neoplasm	I	NCT03843359
	E−7766	Lymphoma Advanced solid tumor	I	NCT04144140
	SYNB-1891	Metastatic solid neoplasm lymphoma	I	NCT04167137
	TAK-676	Non-small cell lung carcinoma Triple-negative breast neoplasm Head and Neck squamous cell carcinoma	I	NCT04879849
		Solid neoplasm	I	NCT04420884
	MK-2118	Lymphoma	I	NCT03249792
	IMSA101	Solid tumor	I/II	NCT04020185
	CDK-002	Advanced solid tumor	I/II	NCT04592484
	MK-1454	Head and neck squamous cell carcinoma	II	NCT04220866
	NOX66	Late-stage prostate cancer	I	NCT03307629
		Metastatic soft-tissue sarcoma	I	NCT05100628
		Metastatic castration-resistant prostate cancer	I/II	NCT04957290
	SB-11285	Melanoma Head and neck squamous cell carcinoma	I	NCT04096638
